# Engineering *Ashbya gossypii* strains for *de novo* lipid production using industrial by‐products

**DOI:** 10.1111/1751-7915.12487

**Published:** 2016-12-23

**Authors:** Patricia Lozano‐Martínez, Rubén M Buey, Rodrigo Ledesma‐Amaro, Alberto Jiménez, José Luis Revuelta

**Affiliations:** ^1^ Metabolic Engineering GroupDepartamento de Microbiología y GenéticaUniversidad de SalamancaEdificio DepartamentalCampus Miguel de Unamuno37007SalamancaSpain; ^2^ Present address: Micalis InstituteINRA UMR1319AgroParisTechUniversité Paris‐Saclay78350Jouy‐en‐JosasFrance

## Abstract

*Ashbya gossypii* is a filamentous fungus that naturally overproduces riboflavin, and it is currently exploited for the industrial production of this vitamin. The utilization of *A. gossypii* for biotechnological applications presents important advantages such as the utilization of low‐cost culture media, inexpensive downstream processing and a wide range of molecular tools for genetic manipulation, thus making *A. gossypii* a valuable biotechnological chassis for metabolic engineering. *A. gossypii* has been shown to accumulate high levels of lipids in oil‐based culture media; however, the lipid biosynthesis capacity is rather limited when grown in sugar‐based culture media. In this study, by altering the fatty acyl‐CoA pool and manipulating the regulation of the main ∆9 desaturase gene, we have obtained *A. gossypii* strains with significantly increased (up to fourfold) *de novo* lipid biosynthesis using glucose as the only carbon source in the fermentation broth. Moreover, these strains were efficient biocatalysts for the conversion of carbohydrates from sugarcane molasses to biolipids, able to accumulate lipids up to 25% of its cell dry weight. Our results represent a proof of principle showing the promising potential of *A. gossypii* as a competitive microorganism for industrial biolipid production using cost‐effective feed stocks.

## Introduction

During the last years, studies unravelling the lipid metabolic pathways in microorganisms have boosted the application of systems metabolic engineering to produce biolipids that could be used to produce biofuels and oleochemicals (Beopoulos *et al*., 2011). Biolipid‐derived fuels from renewable or even waste feedstocks and advanced biofuels avoid the severe inconveniences of first‐ and second‐generation biofuels such as competition with food industry, dependence on climate season and longer processing cycle (Stephanopoulos, 2007; Beopoulos *et al*., 2011; Peralta‐Yahya *et al*., 2012). Biolipids provide a sustainable alternative for fossil fuels that might help to reduce the carbon footprint. Additionally, bio‐based oleochemicals could substitute petroleum‐based chemically synthesized compounds with interest in pharma, food and polymer industries (Ledesma‐Amaro *et al*., 2014b). Thus, the combination of an engineered microorganism host and a cost‐effective feedstock is nowadays a major challenge to produce biofuels and oleochemicals in an environmentally and economically feasible manner.

The model microorganism *Saccharomyces cerevisiae* and the oleaginous yeast *Yarrowia lipolytica* have been extensively manipulated by means of rational metabolic engineering approaches to optimize lipid production (Vorapreeda *et al*., 2012; Blazeck *et al*., 2014; Runguphan and Keasling, 2014; Kavšček *et al*., 2015). Other oleaginous microorganisms such as *Rhodosporidium toruloides* have also been studied for fatty acid‐derived products because of its natural ability to accumulate triacylglycerol (Fillet *et al*., 2015).

*Ashbya gossypii* is a filamentous fungus first identified for its natural capacity to overproduce riboflavin (vitamin B_2_) and, currently, more than half of the worldwide riboflavin industrial production relies on *A. gossypii* fermentation (Stahmann *et al*., 2000; Schwechheimer *et al*., 2016). *A. gossypii* is a very convenient fungus for industrial use because it can be readily grown in industrial waste‐based culture media. These media include low‐cost oils (Schwechheimer *et al*., 2016), glycerol (Ribeiro *et al*., 2012) or sucrose (Pridham and Raper, 1950), the main carbon source of sugarcane molasses (Hashizume *et al*., 1966). This and other advantages have stimulated the use of *A. gossypii* not only for industrial scale riboflavin production, but also for nucleoside production (Ledesma‐Amaro *et al*., 2015a; Ledesma‐Amaro *et al*. 2015b) and recombinant protein production (Magalhes *et al*., 2014).

**Figure 1:**
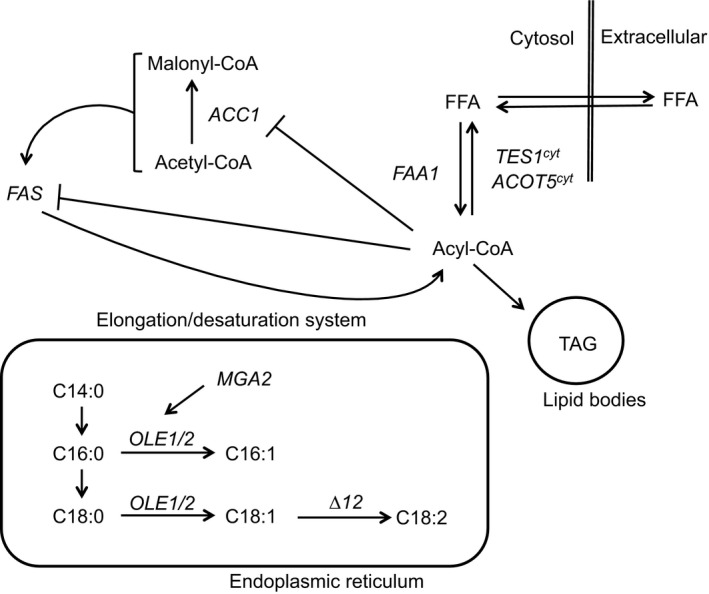
Schematic – simplified – representation of the lipid metabolism in *A. gossypii*. FFA stands for free fatty acids; TAG for triacylglycerol; FAS for fatty acid synthase.

We have previously reported *A. gossypii* strains with compromised lipid β‐oxidation which are able to accumulate lipids up to 70% of the cell dry weight when grown in culture media supplemented with 2% oleic acid. Nevertheless, when grown in glucose‐based media without lipid supplementation, these strains only accumulated up to 10% of their cell dry weight (Ledesma‐Amaro *et al*., 2014a). The development of efficient biocatalyst for the production of biolipids requires not only the conversion of low‐cost oily feedstocks into high‐value oils, but also a high‐yield conversion of carbohydrates to biolipids. In this regard, major bottlenecks exist in the biosynthesis of lipids due to feedback inhibition of lipidogenic enzymes (Fig. 1): acyl‐CoA esters regulate the activity of the fatty acid synthase (FAS), the acetyl‐CoA carboxylase (*ACC1*) and the ∆9 desaturase (*OLE1*; Chen *et al*., 2014; Neess *et al*., 2015). Furthermore, saturated fatty acids also exert a negative effect over the *ACC1* enzyme, thus regulating their own synthesis (Qiao *et al*., 2015).

Here, we aimed at developing of *A. gossypii* strains that significantly increased lipid production using sugar‐based culture media through the manipulation of two major bottlenecks in lipid metabolism: (i) altering the fatty acyl‐CoA pool and the subsequent feedback inhibition of lipidogenic genes and (ii) manipulating the regulation of the main ∆9 desaturase encoded by the *OLE1* gene (Fig. 1). We show that rewiring the regulation of lipogenesis can increase significantly the conversion of carbohydrates to lipids in *A. gossypii*. In addition, we demonstrate that engineered strains of *A. gossypii* are able to produce biolipids when grown on a very simple culture medium consisting of sugarcane molasses and tap water. Our results represent a proof of principle showing the promising potential of *A. gossypii* as a competitive microorganism for industrial biolipid production using cost‐effective feedstocks.

## Results

Aiming at generating *A. gossypii* strains with improved lipid *de novo* biosynthesis, using glucose as the only carbon source, we have manipulated two known bottlenecks for lipid metabolism (Fig. 1): (i) insertion of the first double bond in palmitic and stearic acid by ∆9 desaturases and (ii) alteration of the intracellular concentration of the fatty acyl‐CoA pool.

### OLE1 endogenous regulation is a bottleneck for the *de novo* lipid biosynthesis in *A. gossypii*

*Ashbya gossypii* has two identified and characterized ∆9 desaturases codified by the genes *AgOLE1* and *AgOLE2* that are responsible for the insertion of the first desaturation in stearic and palmitic acid (Fig. 1; Lozano‐Martínez *et al*., 2016). The simultaneous overexpression of these two genes in *A. gossypii* only slightly increased total fatty acid accumulation in glucose‐based medium (up to 1.2‐fold with respect to wild type; Lozano‐Martínez *et al*., 2016). Thereby, contrary to what has been reported for *Y. lipolytica* (Qiao *et al*., 2015), the overexpression of *AgOLE1/OLE2* is not enough to significantly improve the lipid *de novo* biosynthesis in *A. gossypii*. We then decided to study *AgOLE1* regulation.

In *S. cerevisiae*,* MGA2* – and its homologous *SPT23 –* are the main regulators of *OLE1*: Mga2p has been shown not only to activate *OLE1* transcription (Zhang *et al*., 1999; Jiang *et al*., 2001, 2002; Auld *et al*., 2006) but also to stabilize *OLE1 mRNA* transcript when the cells are grown in fatty acid free medium and destabilize it when the cells are exposed to unsaturated fatty acids (Kandasamy *et al*., 2004). *MGA2* codifies for an endoplasmic reticulum membrane protein (Mga2p), but the C‐terminal proteolytic cleavage converts Mga2p into a cytoplasmic protein that can be transported to the nucleus (Martin *et al*., 2007; Liu *et al*., 2015). The deletion of *MGA2* in *S. cerevisiae* has modest effect on cell fitness, but the double knockout of *MGA2* and its homologous *SPT23* results in an inviable mutant in the absence of unsaturated fatty acids in the culture medium (Zhang *et al*., 1999). The overexpression of the cleaved version of *MGA2* in *S. cerevisiae* led to a 1.2‐fold increase in triacylglycerides with respect to wild type (Kaliszewski *et al*., 2008), most probably due to *OLE1* overexpression (Chellappa *et al*., 2001).

Prompted by these results, we decided to investigate the effect on lipid accumulation of the manipulation of the gene *ACR165W* (*AgMGA2*), the Ashbya's homologue of S. cerevisiae *MGA2/SPT23* (Dietrich *et al*., 2004). In glucose‐based media, *AgMGA2* disruption (*mga2Δ*) caused a slight increase in total lipid accumulation, in contrast to its overexpression (*P*_*GPD*_ *‐MGA2*) that showed a significant increase with respect to the wild‐type strain (Table 1). These results agree with previous reports in *S. cerevisiae*, where the *MGA2* orthologues stabilize *OLE1 mRNA* transcript when the cells are grown in fatty acid free medium (Kandasamy *et al*., 2004). On the other hand, *MGA2* might destabilize *OLE1 mRNA* transcript when the cells are exposed to unsaturated fatty acids, as reported for *S. cerevisiae* (Kandasamy *et al*., 2004), which would result in decreased lipid accumulation (Table 1).

**Table 1:** Total fatty acids (TFA) in the engineered *A. gossypii* strains expressed as the percentage of lipids with respect to dry cell weight. Cultures were grown in MA2 media supplemented with either 8% (w/v) Glucose (MA2‐8G) or 1% (w/v) Glucose + 2% (w/v) Oleic Acid (MA2‐1G‐2O) at 28°C for 7 days in an orbital shaker (150 r.p.m.). Numbers are the mean ± SD of two independent experiments with two replicates each. Total biomass showed no large differences among the different strains tested in this study (8.9 ± 2 mg ml^−1^ in MA2‐8G media)

Strain	TFA (%), MA2‐8G	TFA (%), MA2‐1G‐2O
Wild type	5.30 ± 0.4	23.53 ± 0.4
*P*_*GPD*_ *‐MGA2*	8.62 ± 0.3	15.61 ± 2.1
*ΔMGA2*	6.63 ± 0.1	24.08 ± 1.5
*MGA2‐ΔC‐term*	10.47 ± 0.1	24.63 ± 0.9
*P*_*GPD*_ *‐FAA1*	5.90 ± 0.9	11.55 ± 1.9
*ΔFAA1*	2.17 ± 0.4	12.88 ± 2.1
*P*_*GPD*_ *‐TES1* ^*cyt*^	7.89 ± 0.4	n.d.
*P*_*GPD*_ *‐ACOT5* ^*cyt*^	9.85 ± 0.1	n.d.
*MGA2‐ΔC‐term/P*_*GPD*_ *‐ACOT5* ^*cyt*^	20.01 ± 0.2	n.d.

Interestingly, *AgMGA2* disruption does not confer auxotrophy for unsaturated fatty acids (not shown), in contrast to what has been described for *S. cerevisiae* (Kandasamy *et al*., 2004). This might indicate that *MGA2* might be required for maximal transcriptional activation of *OLE1/2*, but it is possible that *OLE1/2* might be expressed at basal levels in the absence of *MGA1*. In this context, *MGA2* deletion would not confer auxotrophy for unsaturated fatty acids. At this point, we do not know if there are additional genes implied in *OLE1/2* transcriptional activation, but no additional paralogues of *MGA2* exist in the genome of *A. gossypii*.

The increase in lipid accumulation was significantly higher when a C‐terminal truncated version of *AgMGA2* was expressed (*mga2‐ΔC‐term*), reaching more than 10% of the cell dry weight (Table 1), similarly to what has been reported for *S. cerevisiae* (Kaliszewski *et al*., 2008). Remarkably, in media supplemented with oleic acid, *P*_*GPD*_ *‐MGA2* strains showed a strong decrease in total lipid accumulation with respect to the wild type, in contrast to both the *mga2Δ* and *mga2‐ΔC‐term* strains that showed no significant differences with the wild type (Table 1).

### The fatty acyl‐CoA pool is a bottleneck for the *de novo* lipid biosynthesis in *A. gossypii*

We next decided to study how the alteration of the fatty acyl‐CoA pool could influence the *de novo* lipid biosynthesis in *A. gossypii*. The intracellular acyl‐CoA pool is extensively regulated by the counteraction of acyl‐CoA synthetases and acyl‐CoA thioesterases (Black and DiRusso, 2007; Chen *et al*., 2014). Acyl‐CoA‐thioesterases catalyse the conversion of activated fatty acids into free fatty acids, which is the reverse reaction catalysed by fatty acyl‐CoA synthetases (Fig. 1).

We first disrupted the main fatty acyl‐CoA synthetase in *A. gossypii*:* AgFAA1*, which is the homolog of *FAA1* and *FAA4* in *S. cerevisiae*. Our results showed that *AgFAA1* is essential when the only carbon source in the culture medium is oleic acid (data not shown) and its disruption (*faa1∆* strain) significantly decreased lipid accumulation as compared to the wild type when grown on glucose‐containing media (Table 1). This finding further supports that *AgFAA1* is the main acyl‐CoA synthetase in *A. gossypii*, in agreement with the phenotype of the *S. cerevisiae faa1∆/faa4∆* strain, which is also unable to grow in fatty acid‐based media (Black and DiRusso, 2007). However, the marked decrease in lipid accumulation in the strain *faa1∆* in glucose‐based media differs from previous results reported for *S. cerevisiae*, where lipid accumulation was not significantly changed with respect to wild type (Færgeman *et al*., 2001; Black and DiRusso, 2007; Chen *et al*., 2014). On the other hand, our results agree with the sharp decrease observed when the strain *faa1∆* of *Y. lipolytica* is grown on oleic acid‐containing media (Dulermo *et al*., 2015). This might indicate that *AgFAA1* has additional functions, apart from the one shown in Fig. 1, in the lipid metabolism of *A. gossypii* that remain unknown. Interestingly, *AgFAA1* overexpression (*P*_*GPD*_ *‐FAA1*) also decreased lipid accumulation in *A. gossypii* when the strain is grown in media containing 2% of oleic acid, despite it has no significant effects in glucose‐based media (Table 1). This might happen because in media‐containing oleic acid *FAA1* overexpression might greatly increase the cytoplasmic levels of fatty acyl‐CoA that would result in the inhibition of lipidogenic genes and, therefore, the observed decrease in lipid accumulation. Moreover, this result agrees with previous reports describing that the alteration of the intracellular acyl‐CoA pool has an inhibitory effect on lipidogenic genes (Færgeman and Knudsen, 1997) and also induced the expression of the genes involved in lipid degradation (Færgeman *et al*., 2001).

We then intended to alter the fatty acyl‐CoA pool by the overexpression of acyl‐CoA‐thioesterase genes. Acyl‐CoA esters are known to repress fatty acid synthesis (Fig. 1) by inhibiting several lipidogenic enzymes, such as *FAS*,* ACC1* and *OLE1* (Bortz and Lynen, 1963; Sumper and Träuble, 1973; Choi *et al*., 1996; Færgeman and Knudsen, 1997). Thereby, we hypothesized that the decrease in the fatty acyl‐CoA intracellular pool (by conversion to free fatty acids) could upregulate lipid accumulation. To test this hypothesis, we constructed two *A. gossypii* strains that ectopically overexpress in their cytoplasm two peroxisomal acyl‐CoA thioesterase enzymes: (i) *P*_*GPD*_ *‐TES1* ^*cyt*^ that overexpress *A. gossypii TES1* (the homologue of *TES1* in *S. cerevisiae*) and (ii) *P*_*GPD*_ *‐ACOT5* ^cyt^ that overexpress *Mus musculus ACOT5*, which has been previously shown to increase the accumulation of free fatty acids in *S. cerevisiae* (Chen *et al*., 2014). Both genes encode fatty acyl‐CoA thioesterases involved in fatty acid degradation in the peroxisome (Jones and Gould, 2000; Westin *et al*., 2004; Maeda *et al*., 2006; Chen *et al*., 2014) and contain the ‘SKL’ prototypical C‐terminal peroxisomal targeting signal (PTS) that it is both necessary and sufficient for directing cytosolic proteins to peroxisomes (Gould *et al*., 1987). Interestingly, total fatty acid quantification of both strains grown in glucose‐based media showed a significant increase (up to twofold) with respect to the wild type (Table 1), suggesting that an excess of cytoplasmic fatty acyl‐CoA thioesterase activity results in a decreased acyl‐CoA pool that relieves the feedback inhibition of lipidogenic genes (Fig. 1). The excess of free fatty acids in the cytoplasm can be readily excreted to the culture media and, accordingly, the two engineered strains excreted approximately fivefold more fatty acids (118.25 ± 0.4 and 122.38 ± 0.4 mg l^−1^ for *P*_*GPD*_ *‐TES1* ^*cyt*^ and *P*_*GPD*_ *‐ACOT5* ^*cyt*^ respectively) than the wild type (22.00 ± 0.6 mg l^−1^).

We next combined the two most favourable modifications (Table 1) into a single strain (*MGA2‐ΔC‐term*/*P*_*GPD*_ *‐ACOT5* ^cyt^) and observed an additive effect of both modifications (Table 1). Remarkably, this *A. gossypii* strain accumulates more than 20% of its dry cell weight.

### Sugarcane molasses are a very convenient carbon source for biolipid production in *A. gossypii*

The results obtained with the strain *MGA2‐ΔC‐term*/*P*_*GPD*_ *‐ACOT5* ^cyt^ together with the advantages for industrial use convert *A. gossypii* into a very promising microorganism for biolipid production using sugar‐based culture media formulations. We therefore studied the use of convenient culture media for industrial use. To this end, we tested sugarcane molasses as the unique carbon source for the culture media of *A. gossypii*.

Molasses, from sugarcane or beet, mainly contains fructose, sucrose and glucose. This sugar is an industrial by‐product from sugar manufacturing, and it is considered an ideal raw material for cheap medium culture formulations (Chao *et al*., 2013). Indeed, sugarcane molasses have been previously proved to be acceptable carbon sources for lipid production in *Y. lipolytica* (Gadjos *et al*., 2015), as well as for ethanol and butanol production in *S. cerevisiae* (Ni *et al*., 2012; Arshad *et al*., 2014).

**Figure 2:**
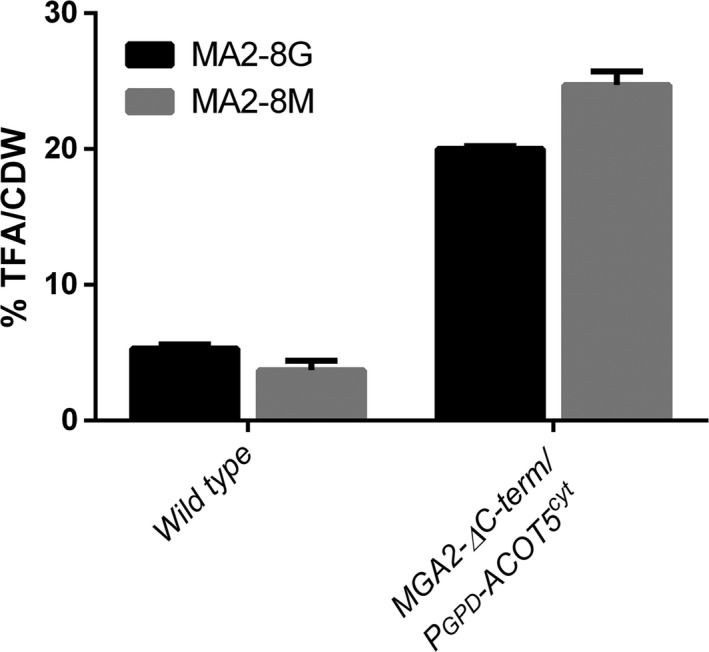
Comparison of total fatty acid (TFA) per cell dry weight (CDW) in the wild‐type and the *MGA2‐ΔC‐term*/*P*_*GPD*_ *‐ACOT5* ^cyt^ strains. MGA2‐8G and MGA2‐8M stand for MA2 medium supplemented with 8% (w/v) of either glucose or sugarcane molasses respectively. The data shown represent the mean of three independent experiments with standard errors.

**Figure 3:**
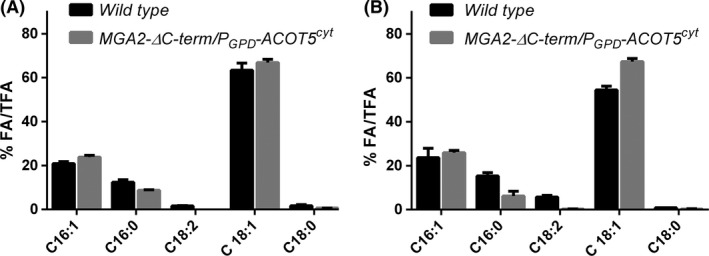
Lipid profile in the wild‐type and the *MGA2‐ΔC‐term*/*P*_*GPD*_ *‐ACOT5* ^cyt^ *A. gossypii* strains in MGA2‐8G (A; MA2 medium with 8% (w/v) glucose) and MGA2‐8M (B; MA2 medium with 8% (w/v) sugarcane molasses). The data shown represent the mean of three independent experiments with standard errors.

Remarkably, in contrast to the wild‐type strain which slightly decreased lipid accumulation in molasses with respect to glucose‐based medium, the *MGA2‐ΔC‐term*/*P*_*GPD*_ *‐ACOT5* ^cyt^ *A. gossypii* engineered strain increased lipid accumulation up to 25% of its dry cell weight (Fig. 2). We then quantitatively characterized the lipid profile of the engineered strain when grown in glucose and molasses‐based culture media. As can be observed in Fig. 3, there are no significant changes between both media. The strain *MGA2‐ΔC‐term*/*P*_*GPD*_ *‐ACOT5* ^cyt^ showed a slight increase in saturated C16:1 and C18:1, in detriment of C16:0 and C18:0 fatty acids (Fig. 3), as an expected consequence of the upregulation of the Δ9 desaturase *AgOLE1*.

Altogether, by manipulating two genes, we have obtained an *A. gossypii* strain able to accumulate up to 25% of its dry cell weight in a very convenient culture media composed of sugarcane molasses and tap water. Furthermore, we envisage that this number can be readily increased through a systematic optimization of the culture medium composition as well as the fermentation conditions. Eventually, further genetic manipulations of this strain by means of random and/or rational modifications could also increase this number.

## Discussion

*Ashbya gossypii* has a large capacity to accumulate lipids when grown in media‐containing oleic acid (Ledesma‐Amaro *et al*., 2014a). Encouraged by this result, we aimed at further manipulating *A. gossypii* to optimize the *de novo* lipid biosynthesis and have obtained strains with increased *de novo* lipid biosynthesis, rather than lipid accumulation. These manipulations enable the utilization of *A. gossypii* as an efficient biocatalyst for the production of biolipids from sugar‐based by‐products such as molasses. The most productive strain contained only two modifications that resulted in additive effects on lipid accumulation: a truncated version of *MGA2*, a main regulator of *OLE1* (the main ∆9 desaturase in *A. gossypii*) and a heterologously expressed murine thioesterase gene, *MmACOT5*. These modifications are expected to increase the expression of *OLE1* and promote the lipid biosynthetic process. In addition, the expression of a heterologous thioesterase is expected to decrease the cytoplasmic pool of fatty acyl‐CoA, thus alleviating the feedback inhibition mechanisms of that this metabolite exerts on lipogenesis. Up to our knowledge, this is the first report on the combination of the manipulation of these two genes in microorganisms to enhance *de novo* lipid biosynthesis. We envisage that further manipulations will readily increase the percentage of lipid accumulation and, indeed, significant efforts are being directed at present in our laboratory towards this aim.

Although the achieved amount of lipid accumulation is not as high as that reported for *S. cerevisiae* and/or *Y. lipolytica* (Kamisaka *et al*., 2013; Blazeck *et al*., 2014), it must be stressed here that *A. gossypii* shows important advantages for industrial production of lipids compared to these yeasts that make of it a promising competitive candidate to be taken into account. First, the biomass from a filamentous fungus can be easily separated from culture media by convenient filtration or sedimentation techniques, easy to implement at industrial level (Zheng *et al*.,^2012^). Second, large‐scale fermentations are nowadays used for riboflavin production, demonstrating the suitability of this fungus for industrial scale‐up. Third, *A. gossypii* hyphae suffer autolysis in the late stationary phase, and triglycerides could be easily recovered by centrifugation, avoiding costly cell‐disruption processes. Therefore, our results represent a proof of principle showing that *A. gossypii* is a promising and convenient microorganism that deserves the further investigation of its potential use as a convenient industrial biolipid producer.

On the other hand, one of the disadvantages of using microbial hosts for lipid production is the global cost of the process, which can be notably diminished with the use of alternative feed stocks such as industrial by‐products. Thereby, the efficient utilization of alternative sources of carbon will have important economic advantages for the scale‐up of lipid production with *A. gossypii* using a cheap and convenient culture media. Sugarcane molasses represent a cheap industrial by‐product consisting on sucrose (up to 50%), nitrogen source, proteins, vitamins and amino acids among others. The use of molasses presents important advantages with respect to other waste products such as lignocellulosic biomass, which needs a costly pre‐treatment for its consumption by microorganisms (Stephanopoulos, 2007; Taherzadeh and Karimi, 2008). Interestingly, *A. gossypii* can grow on molasses without any further modification, contrary to what happens in *Y. lipolytica* that needs the ectopic overexpression of invertases to degrade sucrose (Gadjos *et al*., 2015). Thereby, the engineered *MGA2‐ΔC‐term*/*P*_*GPD*_ *‐ACOT5* ^cyt^ strain is a promising candidate that deserves future attention.

The lipid profile of the engineered oleaginous strain has a composition in monounsaturated, polyunsaturated and saturated methyl esters that correlate with good biodiesel properties, that is neither high levels of polyunsaturated nor long‐chain saturated FAs (Ramos *et al*.,^2009^). Thereby, this strain accumulates significant amounts of lipids suitable for biodiesel production. Furthermore, we have recently reported that the modification of the lipid profile by manipulating the elongation and desaturation systems enhances biodiesel properties in *A. gossypii* (Ledesma‐Amaro *et al*., 2014b). Thus, future experiments in our laboratory will be focused on the modification of the lipid profile in our engineered oleaginous strain.

Altogether, our results demonstrate that *A. gossypii* is a very promising industrial microorganism that uses a cost‐effective feedstock to *de novo* synthetize significant amounts of biolipids that can be used for producing biofuels in an environmentally and economically feasible manner.

## Experimental procedures

### *A. gossypii* strains, media and growth conditions

The *A. gossypii* strain ATCC10895 was used and considered wild‐type strain. The strains were cultured at 28°C using MA2 rich medium during 7 days (Förster *et al*., 1999). MA2 is composed of yeast extract, bacto‐peptone, agar, water and glucose. In this study, for lipid accumulation, the C/N ratio was increased using 8% of glucose (MA2‐8G) as carbon source instead of 2%, which is the standard formulation. Alternatively, 1% glucose and 2% oleic acid, previously emulsified by sonication in the presence of 0.02% Tween‐40 (MA2‐1G‐2O), were used. For experiments with molasses, media was prepared with 8% sugarcane molasses (kindly provided by AB Azucarera Iberia S.L.), 0.1% of yeast extract and tap water (MA2‐8M). *A. gossypii* transformation, sporulation conditions and spore isolation have been described elsewhere (Santos *et al*., 2005). Briefly, DNA was introduced into *A. gossypii* by electroporation, and primary transformants were isolated in selective medium. Homokaryon transformant clones were obtained by sporulation of the primary heterokaryon transformants and isolated on antibiotic‐containing plates with 250 mg l^−1^ of geneticin (*G418*). Liquid cultures were initiated from spores and were incubated on an orbital shaker at 200 r.p.m at 28°C.

### Gene manipulation of *A. gossypii*

Gene deletion and overexpression were carried out by the construction of recombinant integrative cassettes (Ledesma‐Amaro *et al*., 2014a,b). For gene deletion, a replacement cassette with selection marker (*loxP‐KanMX‐loxP* module for *G418* resistance) was used. This selection marker is flanked by the repeated inverted sequences *loxP,* which enable the elimination of the selection marker by the expression of a Cre recombinase (Ledesma‐Amaro *et al*., 2014a). The deletion of the C‐terminal part of *AgMGA2* was performed by substituting this region by a *G418* antibiotic resistance marker. For gene overexpression, a module based on the *A. gossypii* glycerol 3‐phosphate dehydrogenase promoter (*P*_*GPD*_) and phosphoglycerate kinase (*T*_*PGK1*_ *)* terminator sequences, recombinogenic flanks and the antibiotic selectable marker *loxP‐KanMX‐loxP,* was integrated at the *STE12* locus. DNA constructs were obtained using Golden Gate methodology (Enger *et al*., 2008). Genome integration of the deletion and overexpression modules was confirmed by analytical PCR and DNA sequencing.

To ectopically express *TES1* and *ACOT5* in the cytosol of *A. gossypii*, we removed the C‐terminal prototypical peroxisomal targeting signal (PTS) that it is both necessary and sufficient for directing cytosolic proteins to peroxisomes (Gould *et al*., 1987). The signal ‘SKL’ that both *TES1* and *ACOT5* contain is a prototypical PTS and, thereby, its removal avoids peroxisome localization.

### Lipid extraction and quantification

Triacylglycerols were extracted and trans‐methylated from lyophilized biomass using a modification of the method described by Bligh and Dyer (Bligh and Dyer, 1959). Approximately 200 μg of dried mycelia was mixed with 1 ml of 97.5% methanol/2.5% sulfuric acid and incubated at 80°C for 90 min. The transesterification reaction was stopped by the addition of 1 ml of distilled water. The extraction was performed by mixing the samples with 0.5 ml of hexane and recovery of the upper phase after centrifugation. The hexane‐soluble extracted fatty acid methyl esters dissolved were analysed by gas chromatography coupled to mass spectrometry (GC‐MS) in an Agilent 7890A gas chromatograph coupled an Agilent MS200 (Agilent, Santa Clara, California, USA) mass spectrometer. A VF50 column (30 m long, 0.25 mm internal diameter and 25 μm film) was used using helium as carrier at 1 ml min^−1^, with a split ratio of 1:20. The oven programme was as follows: an initial temperature of 90°C for 5 min, a ramp of 12°C min^−1^ up to 190°C and a ramp of 4°C min^−1^ up to 290°C. MS detection was from 50 to 400 Da. Fatty acids were identified by comparison with commercial fatty acid methyl ester standards (FAME32; Supelco), and total quantification of fatty acids, expressed as total fatty acids (TFA), was performed using an internal standard: 50 μg of heptadecanoic acid C17:0 (Sigma‐Aldrich, Sigma‐Aldrich Quimica SL, Madrid, Spain).

## Conflict of interest

The authors declare that they have no competing interests.
